# A special type of liver failure after partial hepatectomy: a case report

**DOI:** 10.3389/fonc.2025.1590035

**Published:** 2025-06-02

**Authors:** Linxiao Gao, Bing Tu, Mingxiang Cheng, Menghao Wang

**Affiliations:** Department of Hepatobiliary Surgery, The Second Affiliated Hospital of Chongqing Medical University, Chongqing, China

**Keywords:** hepatocellular carcinoma, hepatectomy, liver failure, liver necrosis, case report

## Abstract

Posthepatectomy liver failure (PHLF) is one of the most harmful complications after liver resection. Here, we report a case of a specific type of PHLF in a 60-year-old man with hepatocellular carcinoma. The patient developed extensive liver necrosis accompanied by further deterioration of liver function and coagulation on the eighth postoperative day. After being treated with liver protection, circulation improvement, plasma infusion, and anti-infective therapy, his bilirubin level still increased progressively, and renal function deteriorated with anuria. Finally, the patient’s family discontinued treatment. This case highlights the importance of the timely identification and management of this special type of PHLF.

## 1 Introduction

Hepatocellular carcinoma (HCC) is the leading cause of cancer-related mortality worldwide (1). Hepatic resection is an established curative treatment for patients with HCC, especially in those with good hepatic reserve function and early-stage tumors (2). Despite advances in liver surgery, posthepatectomy liver failure (PHLF) is still one of the most serious posthepatectomy complications and a major cause of mortality, usually occurring within 5 days after surgery (3), with an incidence of up to 32% (4). PHLF is characterized by fluid retention, coagulopathy, non-obstructive jaundice, and increased infection susceptibility.

Previous studies have attributed PHLF to factors such as hepatitis B virus (HBV) reactivation, liver cirrhosis, intraoperative bleeding, inadequate remnant liver volume, and postoperative portal vein thrombosis (5–7). However, in clinical practice, the above-mentioned factors do not fully account for the etiology of PHLF. Herein, we present a rare case of a special type of PHLF with unknown causes.

## 2 Case presentation

**Figure 1:**
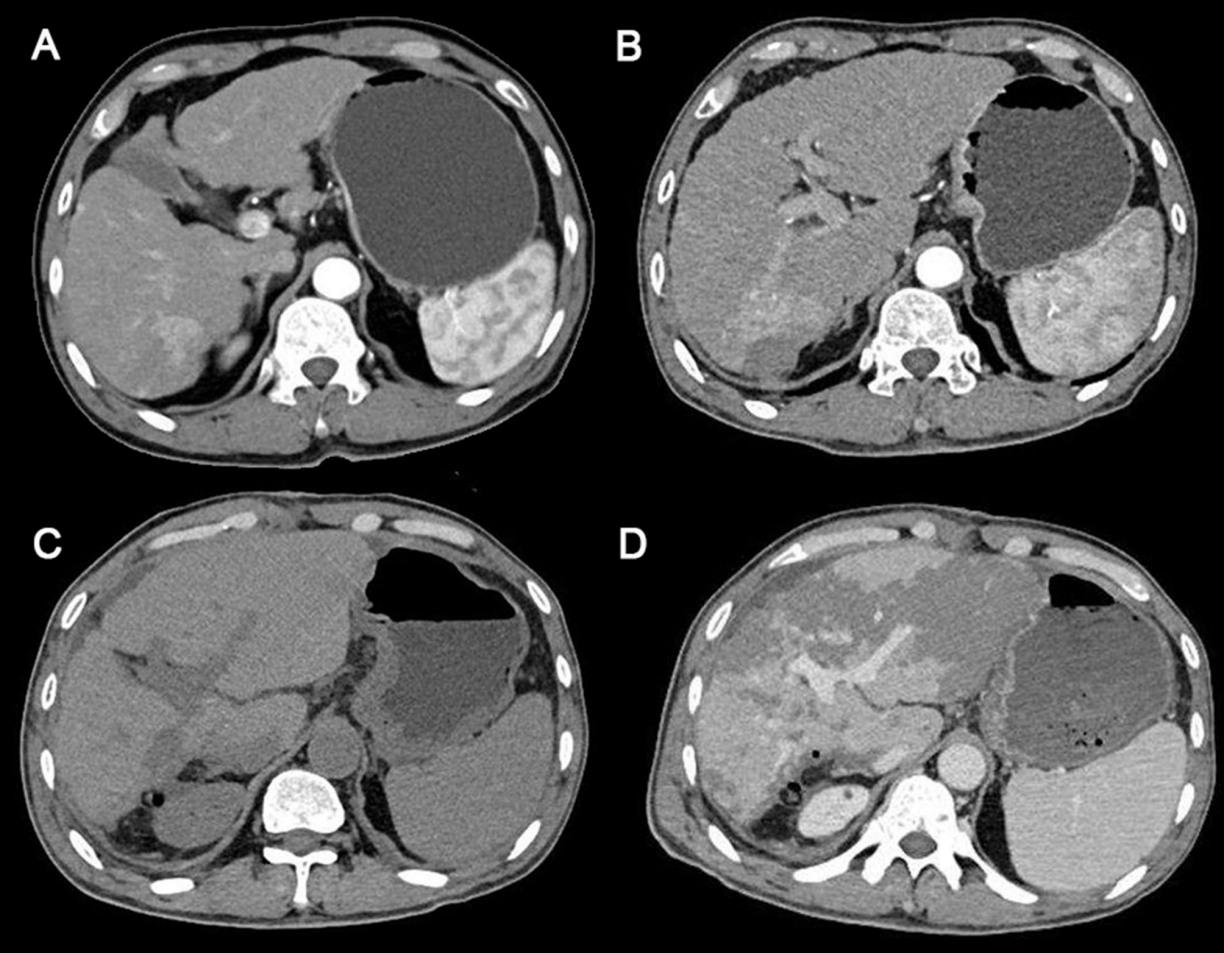
Abdominal computed tomography scans of the patient. **(A)** The time the patient was first diagnosed with HCC 2 years ago. **(B)** Before surgery. **(C)** The sixth day after surgery. **(D)** The eighth day after surgery (the day of PHLF onset). HCC, hepatocellular carcinoma; PHLF, posthepatectomy liver failure.

On February 22, 2024, a 60-year-old man was admitted to the hospital with a recurrence of HCC. The patient, due to upper abdominal pain, presented to the hospital 2 years ago. Abdominal enhanced computed tomography (CT) examination found a 38 × 23 mm nodule located in the right posterior lobe of the liver (**Figure 1A**). The clinical diagnosis was HCC; the patient refused surgery, and percutaneous hepatic puncture radiofrequency ablation was therefore performed. One day before admission, the magnetic resonance imaging examination of the patient in the outpatient clinic showed the recurrence of HCC.

Subsequent laboratory investigations after admission revealed alpha-fetoprotein of 2.83 μg/L (normal, 0–13.2 μg/L), hepatitis B surface antigen (+), and quantification of HBV-DNA (−). Abdominal enhanced CT indicated a possibility of recurrence of hepatocellular carcinoma, with invasion of the right posterior branch of the portal vein and tumor thrombus formation, and intrahepatic bile duct invasion (**Figure 1B**). Therefore, the patient was clinically diagnosed with HCC, Barcelona Clinic Liver Cancer (BCLC) stage A, and T_4_N_0_M_0_. The patient had a regularly treated HBV infection for more than 2 years. The patient had diabetes for 1 year and was taking dapagliflozin regularly for glycemic control. He denied a history of other chronic diseases, such as hypertension and coronary heart disease, and a history of smoking and drinking.

The patient was generally in good condition before surgery, with an Eastern Cooperative Oncology Group Performance Status (ECOG-PS) score of 0 and a Nutritional Risk Screening 2002 (NRS 2002) score of 1. Preoperative comprehensive evaluation of liver reserve function showed the following: Child–Pugh class A, albumin–bilirubin (ALBI) grade score of −3.04 (grade 1), model for end-stage liver disease (MELD) score of 4.88, MELD-Na score of 5.46, standard liver volume (SLV) of 1126.0 cm^3^, indocyanine green 15-minute retention rate (ICG-R15) of 4.9%, liver stiffness value of 20.6 kPa, and the ratio of future liver remnant (FLR) of 84.6%. After fully explaining the patient’s condition and introducing the alternative treatments, such as transarterial chemoembolization, radiofrequency ablation, targeted drug therapy, and immune-checkpoint inhibitor therapy, the patient and his family chose surgery.

The anatomical partial hepatectomy was performed on March 4, 2024. During the operation, the right posterior hepatic pedicle was found along the Laennec membrane. The hepatic parenchyma was severed using an ultrasonic knife and a clamp, and the first portal of the liver was blocked using the Pringle method. If necessary, an intermittent portal of the liver was blocked for a single time of ≤15 minutes, and the total ischemia time was 95 minutes. The duration of the surgery was 572 minutes, and the total blood loss was 1,000 mL. Finally, the right posterior lobe of the liver was anatomically resected. The pathology report confirmed moderate differentiation of hepatocellular carcinoma, the resection margin was negative, and no cancerous tissue was found. Cefmetazole was routinely used after the operation to prevent infection. Postoperative blood tests indicated that the patient’s aminotransferase level increased. Anticoagulant therapy (fondaparinux, 1.25 mg QD) was started on the second day after surgery to prevent thrombosis in the deep veins and portal veins. During anticoagulation, the prothrombin time, prothrombin activation, and the international normalized ratio were continuously monitored. A routine follow-up abdominal CT on March 10 showed no significant abnormalities (**Figure 1C**). Liver function improved after liver protection treatment.

**Figure 2:**
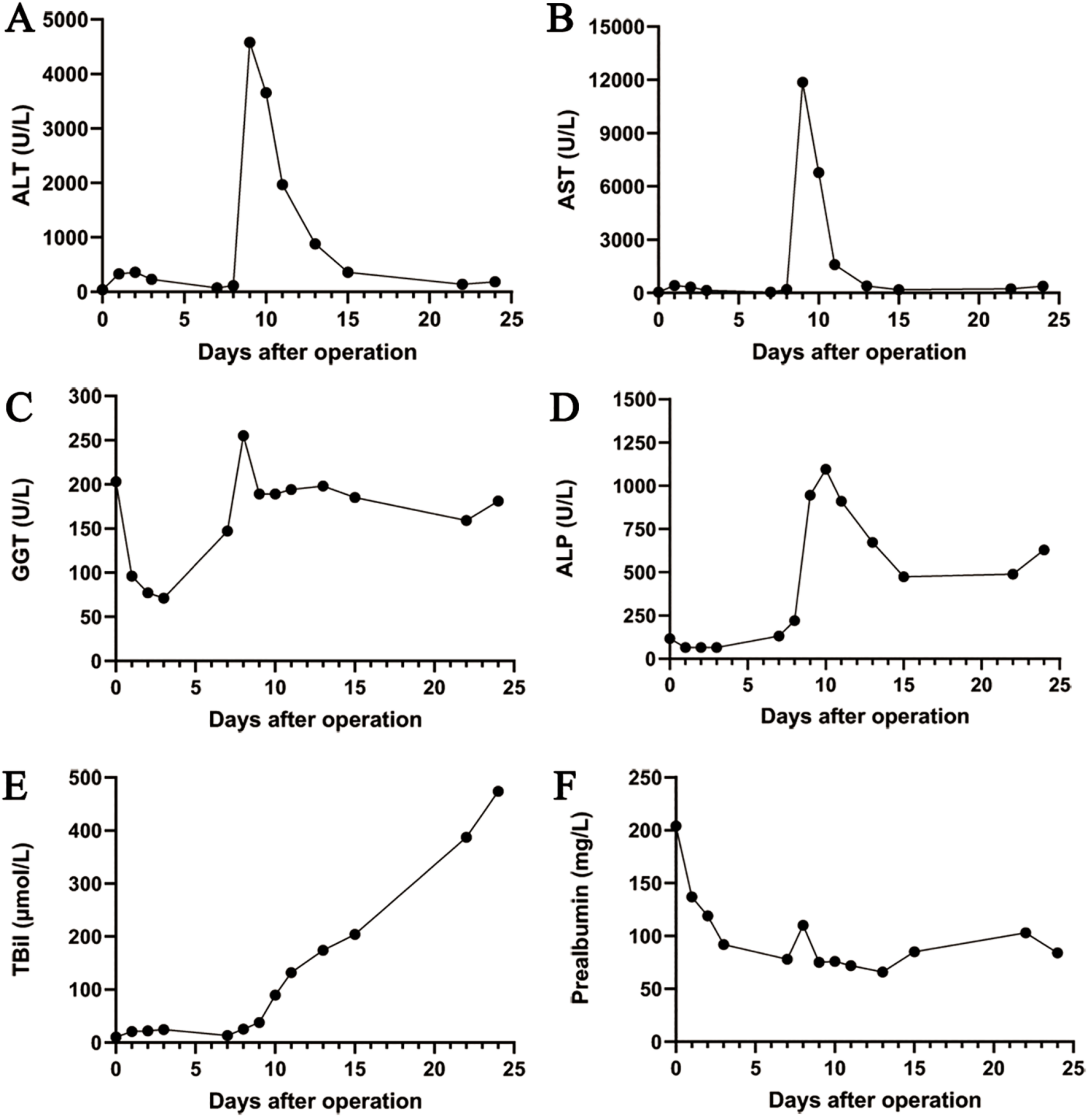
The trend chart of changes in the liver function. **(A)** ALT. **(B)** AST. **(C)** GGT. **(D)** ALP. **(E)** TBil. **(F)** Prealbumin. ALT, alanine aminotransferase; AST, aspartate aminotransferase; GGT, gamma-glutamyl transferase; ALP, alkaline phosphatase; TBil, total bilirubin.

**Figure 3:**
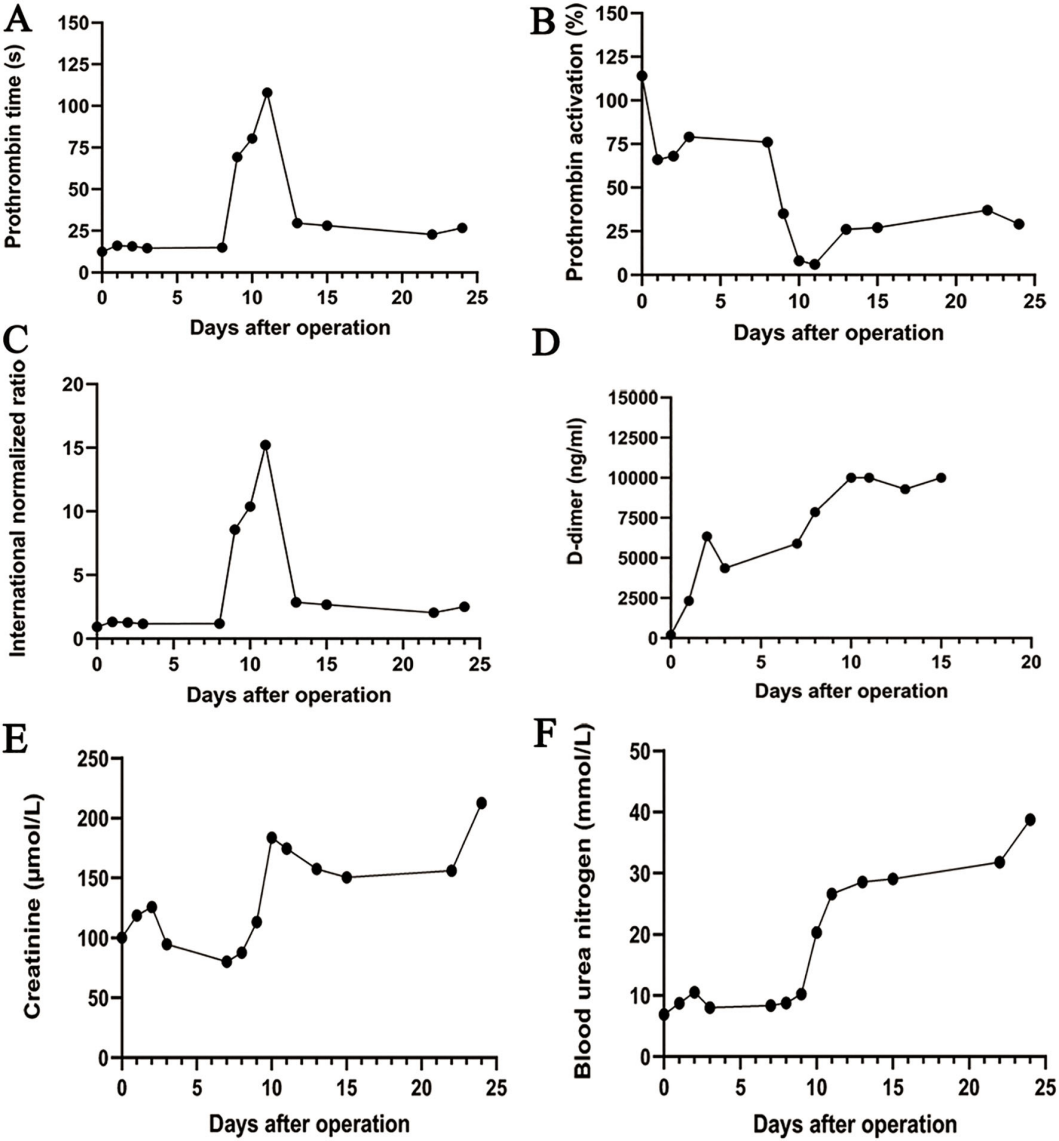
The trend chart of changes in the coagulation and renal function. **(A)** Prothrombin time. **(B)** Prothrombin activation. **(C)** International normalized ratio. **(D)** D-dimer. **(E)** Creatinine. **(F)** Blood urea nitrogen.

On March 12, 2024, the eighth day after surgery, the patient suddenly developed severe abdominal pain. Imaging studies revealed few new flaky, low-density, and unenhanced shadows in the residual liver, considered as acute liver necrosis (**Figure 1D**). Abdominal ultrasonography showed no thrombus formation in hepatic vessels, and no evident signs of ischemia were observed in the portal vein or hepatic arterial/venous circulation. Given the hypothesis of hepatic microcirculation thrombosis, anticoagulation was switched to rivaroxaban (1.5 mg QD). The next day, the patient developed a fever with a peak temperature of 38.7°C. Blood cultures were negative, while ascitic fluid cultures revealed infection with *Staphylococcus haemolyticus* and *Enterococcus faecalis*. Procalcitonin levels were significantly elevated (2.83 ng/mL) compared to the value of the previous day (0.71 ng/mL). Consequently, antibiotic therapy was escalated to imipenem. In the following few days, blood tests showed that the patient’s transaminase level increased sharply, with a progressive rise of bilirubin, continuous deterioration of clotting function, reactivation of HBV-DNA, and elevation of blood ammonia (**Figures 2**, **3**). Consequently, rivaroxaban was discontinued on March 15 due to coagulopathy.

**Figure 4:**
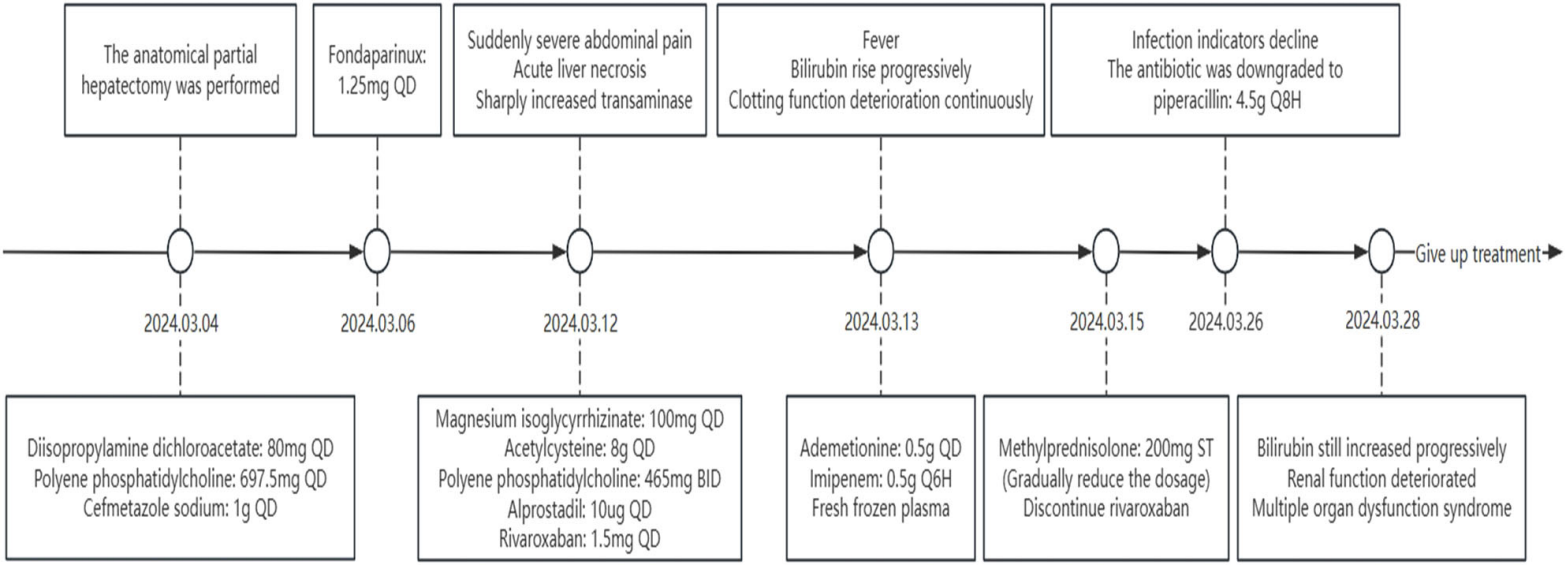
Timeline of treatment.

After being treated with liver protection (polyene phosphatidylcholine, membrane stabilization and hepatocyte regeneration, magnesium isoglycyrrhizinate: anti-inflammatory, acetylcysteine: free radical scavenger, and ademetionine: methylation regulator and detox booster), circulation improvement (rivaroxaban, alprostadil, and papaverine), plasma infusion, and anti-infective therapy (imipenem), the patient’s bilirubin level still increased progressively, and renal function deteriorated with anuria (**Figure 4**). On March 26, the patient’s infection markers decreased compared to prior measurements, and no recurrent fever was observed. Antibiotic therapy was therefore de-escalated to piperacillin. Despite prompt intervention, the disease progressed rapidly. Regrettably, the patient’s family decided to discontinue treatment on March 28, 2024, due to the presence of multiple organ dysfunction syndrome. Subsequent follow-up indicated that the patient passed away post-discharge.

## 3 Discussion

The International Study Group of Liver Surgery (ISGLS) defines PHLF as a decline in the ability of the liver to maintain its synthetic, excretory, and detoxification functions, with an elevated international normalized ratio and hyperbilirubinemia on or after postoperative day 5, in the absence of biliary obstruction (8). Due to its potentially fatal consequences, the prevention and management of PHLF remain major challenges in the surgical treatment of HCC. Several risk factors and parameters, such as biochemical liver function, ICG-R15 tests, and FLR, have been regarded as predictors of PHLF (9–11). However, in this report, no risk factors for PHLF were identified in our patient following preoperative assessments of liver function, ICG-R15, residual liver volume, and so on. Moreover, conventional PHLF does not typically present with large areas of hepatic necrosis on imaging. This suggested a complex pathogenesis underlying PHLF, especially this late-onset PHLF, not easily predicted by a few single indicators. We thus defined a special type of PHLF exhibiting a characteristic aminotransferase trajectory (initial postoperative rise and subsequent decline to near-normal levels, followed by a sudden, sharp re-elevation), delayed-onset total bilirubin increase, abnormal coagulation parameters, and diffuse hepatic necrosis on imaging without evidence of thrombosis. This subtype demonstrates rapid progression and poor prognosis.

The volume of liver resection is the most important factor influencing the occurrence of PHLF. Resection of more than 50% of the liver parenchyma or major hepatectomy is recognized as a risk factor for PHLF (12). Intraoperative portal vein hypertension, intraoperative blood loss greater than 1,200 mL, intraoperative transfusion, prolonged hepatic blood flow occlusion, and extended total surgical duration are also closely associated risk factors for PHLF (13, 14). Previous studies have suggested that hepatitis B patients are more susceptible to PHLF, as HBV-expressing hepatocytes exhibit reduced sensitivity to insulin, impairing liver regeneration (15). Notably, our patient developed reactivation of HBV post-surgery in this case. Additionally, factors such as obesity, malnutrition, smoking, and alcohol consumption can contribute to PHLF by impairing immune function, increasing infection risk, and hindering liver cell regeneration (2). The internationally recognized “50–50 criteria” (prothrombin activation <50% and bilirubin >50 μmol/L on postoperative day 5) currently serve as a simple, early, and accurate predictor of a more than 50% mortality rate after hepatectomy (16). However, in this case, both prothrombin activation and bilirubin levels returned to normal ranges by postoperative day 5. Additionally, the Child–Pugh scoring system and the ALBI scoring system are frequently utilized to evaluate the prognosis after hepatectomy in clinical practice (17). However, this patient’s preoperative prognostic scores likewise indicated low-risk status. Therefore, for this special type of PHLF, conventional risk stratification tools fail to reliably predict outcomes.

At present, the reason for the occurrence of such PHLF is not clear. According to the relevant data of the patient, the preoperative liver reserve function of the patient was good, and the residual liver volume was sufficient. The cause of postoperative liver necrosis also remained unclear, with no evidence of thromboembolism or ischemia identified. Furthermore, the postoperative medications administered (including cefmetazole, dichloroacetate, polyene phosphatidylcholine, and diisopropylamine dichloroacetate) have no established hepatotoxicity, thus excluding drug-induced liver injury as a contributing factor. Furthermore, preoperative evaluations and previous hospital admissions revealed no evidence of autoimmune hepatitis. First, that HBV activity significantly increases the incidence of PHLF could be an important contributing factor, while intraoperative bleeding and surgical stress may trigger HBV reactivation (18). Based on the progression of the condition and postoperative examinations, we hypothesize that there may be other potential causes. Imaging revealed large liver necrotic lesions, while hematological analysis showed a significant increase in D-dimer, possibly due to thrombosis in the hepatic microcirculation. This may also explain the acute abdominal pain preceding disease onset. However, intrahepatic vascular ultrasound did not confirm any flow abnormalities, nor was there any pathological evidence to support this hypothesis. Second, in patients with cirrhosis, gut microbiota translocation can lead to the formation of portal vein system bacterial emboli, which in turn alters the hepatic immune microenvironment and causes hepatic sinusoidal obstruction, ultimately resulting in hepatic ischemic necrosis (19). Third, the patient in this case presented with marked fever, elevated infection markers, and positive ascitic fluid cultures. The inflammatory response plays a pivotal role in modulating hepatic regeneration, which is an essential mechanism for functional recovery posthepatectomy (20). The persistent inflammatory microenvironment has been shown to suppress liver regeneration and accelerate functional failure of remnant liver tissue (21). Moreover, excessive shear stress has been pathophysiologically linked to both endothelial necrosis and oxidative impairment of regenerating hepatocytes. The extensive intra-abdominal adhesions and prolonged operative duration in this case may represent potential contributing factors for PHLF.

The progress of the special type of PHLF is rapid, and the prognosis is poor. The focus of PHLF treatment is to promote hepatocyte regeneration and maintain hepatic physiological function until the liver has sufficiently regenerated to meet its own metabolic demands. Based on the aforementioned cases, for the early detection and management of such type of PHLF, first, we propose that early postoperative anticoagulation and improvement of microcirculation should be emphasized. Second, postoperative liver and coagulation monitoring is critical. Once a sharp increase in transaminase level is detected, an urgent enhanced CT and multidisciplinary intervention are warranted to minimize liver damage and prevent necrosis progression. Third, dynamic monitoring of infection markers is essential postoperatively. Antimicrobial therapy should be strengthened when indicated, along with peritoneal drainage cultures, to assess intra-abdominal infection status and guide antibiotic selection. Additionally, approximately 5 days postoperatively or in cases of PHLF, routine re-assessment of HBV-DNA levels is recommended. Finally, vigilant monitoring and protection of extrahepatic organ function are crucial to optimize hepatorenal circulation and prevent multiple organ dysfunction. While this case provides valuable insights into a special type of PHLF, we acknowledge the inherent limitations of a single-case report. The findings may not be generalizable due to individual variations in pathophysiology and treatment response. Future multicenter studies with larger cohorts are needed to validate our observations.

## 4 Conclusion

In summary, this report describes a specific type of PHLF in a patient with HCC who developed liver failure accompanied by extensive hepatic necrotic lesions following partial hepatectomy. The special type of PHLF in this study has an insidious onset and rapid progress, warranting close attention. Clinicians should remain highly suspicious of the special type of PHLF in high-risk patients.

## Data Availability

The original contributions presented in the study are included in the article/Supplementary Material. Further inquiries can be directed to the corresponding authors.
